# Defining postoperative spinal infections: navigating the inconsistencies in diagnostic definitions

**DOI:** 10.5194/jbji-10-451-2025

**Published:** 2025-11-12

**Authors:** Seyed Mohammad Amin Alavi, Fabio Borgonovo, Francesco Petri, Takahiro Matsuo, Andrea Gori, Jeremy D. Shaw, Aaron J. Tande, Elie F. Berbari

**Affiliations:** 1 Faculty of Medicine, Ahvaz Jundishapur University of Medical Sciences, Ahvaz, Iran; 2 Division of Public Health, Infectious Diseases and Occupational Medicine, Department of Medicine, Mayo Clinic College of Medicine and Science, Mayo Clinic, Rochester, 55905 MN, USA; 3 Department of Infectious Diseases, “Luigi Sacco” University Hospital, ASST FBF Sacco, 20157 Milan, Italy; 4 Department of Biomedical and Clinical Sciences, University of Milan, 20157 Milan, Italy; 5 Intermountain Neurosciences, Intermountain Health, Salt Lake City, UT, USA

## Abstract

The absence of a standardized definition for postoperative spinal infections (PSIs) hinders both diagnosis and research. Using a meta-epidemiological approach, we analyzed 101 studies, with most relying on predefined criteria but with a minority creating their own definition (mainly clinical). Establishing a universal definition is crucial to enhancing PSI management and facilitating research.

## 1 Introduction

The rising number of spine surgeries has resulted in an increased absolute burden of postoperative spinal infections (PSIs), including surgical site infections (SSIs), which are now the third most common complication following spine surgeries (Wang et al., 2022, 2023).

Previous studies have used a range of definitions for PSI, often relying on surveillance-oriented criteria that may not fully capture the clinical complexity of these infections. While the definition provided by the Centers for Disease Control and Prevention (CDC) in conjunction with the National Healthcare Safety Network (NHSN) has contributed to standardized infection surveillance, it is primarily designed for epidemiological monitoring rather than for clinical diagnosis (Tai et al., 2024). Currently, there is no universally accepted definition of PSI. The index procedure may involve instrumentation or be performed without it. Furthermore, infections can affect various anatomical sites, including the vertebrae and intervertebral discs, or may present as abscess formation. This study aimed to identify both clinical diagnostic criteria and surveillance definitions of PSI reported in the literature, using a meta-epidemiological approach. The findings can help guide expert consensus in establishing a shared definition of PSI by addressing the absence thereof, which hinders clinical practice and research by affecting population identification, and by determining desirable outcomes for clinical interventions (Kawtharany et al., 2025).

## 2 Methods

The current study followed Preferred Reporting Items for Systematic Review and Meta-analysis (PRISMA) guidelines adapted for meta-epidemiological methodology (Murad and Wang, 2017). A literature search (Table S1 in the Supplement) was conducted in Ovid MEDLINE (14 March 2024) by a medical librarian, with no date restrictions but limited to English-language human studies. Studies were included if they analyzed at least 10 adults with PSI and provided a clear definition, irrespective of their purpose, either clinical or for surveillance, to ensure generalizability and comprehensiveness. The primary outcome assessed diagnostic criteria of PSIs and their thematic clustering. To standardize the landscape of definitions, 13 predefined criteria for diagnosing PSI were utilized to build combinations, based on clinical practices and prior studies (Table 1). The criteria were as follows: (1) clinical domain – systemic signs and symptoms (e.g., fever, back pain, neurological deficits); (2) clinical domain – secondary wound dehiscence; (3) clinical domain – visible/exposed implant or bone; (4) clinical domain – evidence of intraoperative or wound exudate; (5) clinical domain – inflammatory biomarkers; (6) clinical domain – benefit from conservative or operative treatment; (7) direct evidence domain – any culture from surgery; (8) direct evidence domain – culture from image-guided biopsy; (9) direct evidence domain – blood cultures; (10) direct evidence domain – culture from aspiration fluid, wound culture, or deep swab; (11) direct evidence domain – histopathology; (12) imaging – MRI; and (13) imaging – other than MRI.

## 3 Results

After screening 1466 articles, we included 101 that defined PSI (the PRISMA flowchart is shown in Fig. S1 in the Supplement). Most studies were retrospective (91/101, 92.1 %), with degenerative disorders being the primary indication for spine surgery (24/101, 23.8 %). The sample sizes predominantly ranged from 1000 to 10 000 total patients in each study (46/101, 45.5 %). A pre-established criterion was utilized in 77 out of 101 articles, while 7 out of 101 explicitly cited a criterion with a defined explanation in their article. The most commonly cited criterion was the American College of Surgeons National Surgical Quality Improvement Program (ACS NSQIP) (ACS NSQIP Participant Use Data File, 2025), cited in 53 out of 84 instances (63.1 %). Additionally, 72/101 (71.3 %) studies stratified infections as superficial or deep. Meanwhile, 24 studies provided their specific PSI definition, primarily based on clinical signs and symptoms 11/23 (47.8 %), followed by any cultures from surgery and aspiration fluid, wound culture, or deep swab (both 9/23, 39.1 %) (Table 1). Using the 13 criteria, we were not able to show any clustering of criteria.

**Table 1:** Characteristics of the included studies.

Characteristic	Number of studies ( N=101 )
Study design	
Retrospective	93 (92.1 %)
Prospective	8 (7.9 %)
Indication for surgery	
(1) Trauma	1 (1.0 %)
(2) Cancer	5 (5.0 %)
(3) Infection	2 (2.0 %)
(4) Degenerative	24 (23.8 %)
(5) Deformity	6 (5.9 %)
(6) Other or miscellaneous	31 (30.7 %)
(7) Not specified	32 (31.7 %)
Size of cohort	
< 500 patients	25 (24.7 %)
≥500 – <1000 patients	7 (6.9 %)
≥1000 –10 000 patients	46 (45.5 %)
≥10000 patients	22 (21.8 %)
Type of surgery	
Spinal fusion	49 (48.5 %)
Decompression	33 (32.7 %)
Various types	13 (12.9 %)
Discectomy	5 (4.9 %)
Other	2 (2 %)
Not mentioned	28 (27.7 %)
Definition of SSI	
Cited a reference	77 (76.2 %)
CDC 1992	2/84 (2.4 %)
CDC 1999	12/84 (14.3 %)
CDC 2017	9/84 (10.7 %)
ACS NSQIP	53/84 (63.1 %)
NHSN	0 (0 %)
Other^a^	8/83 (9.6 %)
Author-derived and cited a reference	5 (5 %)
Author-derived	18 (17.8 %)
The definition accounted for the depth of infection (e.g., deep vs. superficial) – yes	72 (71.3 %)
Use of sonication	0 %
Use of molecular methods	0 %
Use of other diagnostic methods (leukocyte esterase, alpha defensin, synovial presepsin, or spine fluid WBC)	0 %
Individual criteria used^b^	
Clinical domain – systemic signs and symptoms (e.g., fever, back pain, neurological deficits)	11/23 (47.8 %)
Clinical domain – secondary wound dehiscence	4/23 (17.4 %)
Clinical domain – visible/exposed implant or bone	3/23 (13.0 %)
Clinical domain – evidence of intraoperative or wound exudate	8/23 (34.8 %)
Clinical domain – inflammatory biomarkers	6/23 (26.1 %)
Clinical domain – benefit from conservative or operative treatment	6/23 (26.1 %)
Direct evidence domain – any culture from surgery	9/23 (39.1 %)
Direct evidence domain – culture from image-guided biopsy	4/23 (17.4 %)
Direct evidence domain – blood cultures	5/23 (21.7 %)
Direct evidence domain – culture from aspiration fluid, wound culture, or deep swab	9/23 (39.1 %)
Direct evidence domain – histopathology	1/23 (4.3 %)
Imaging – MRI	4/23 (17.4 %)
Imaging – other than MRI	4/23 (17.4 %)

^a^ Total can be more than 100 %, since some variables can be present in multiple categories. ^b^ This refers to 23 articles that used a definition derived by the authors. Abbreviations: American College of Surgeons National Surgical Quality Improvement Program (ACS NSQIP), Centers for Disease Control and Prevention (CDC), National Healthcare Safety Network (NHSN), Preferred Reporting Items for Systematic Review and Meta-analysis (PRISMA), magnetic resonance imaging (MRI)

**Figure 1:**
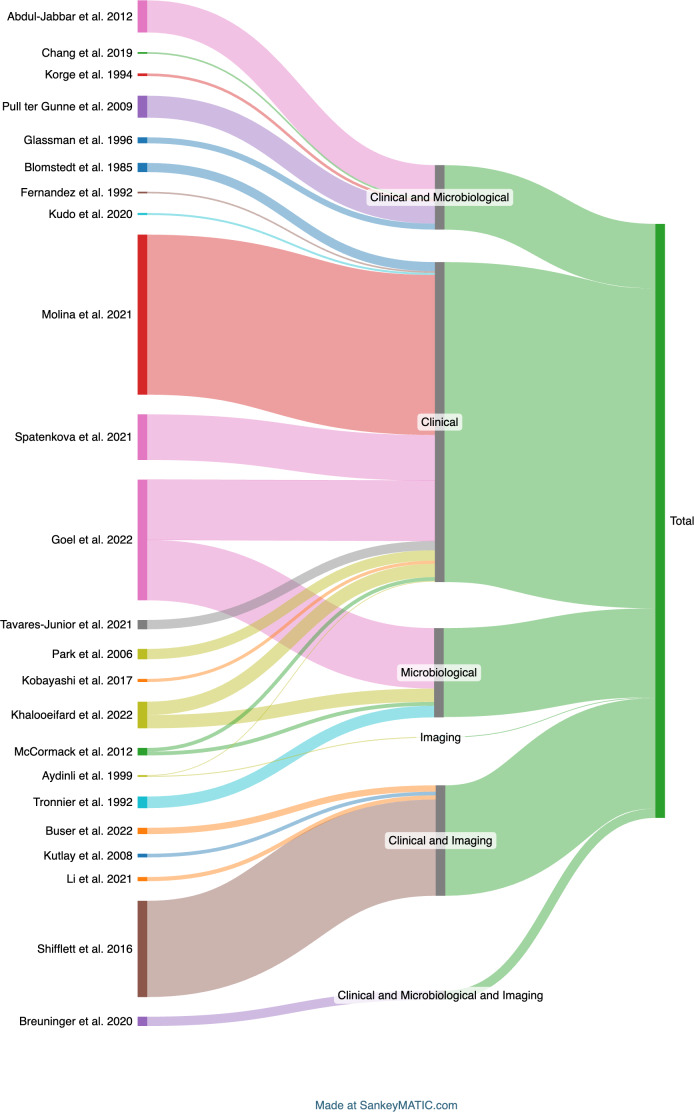
Sankey diagram showing the distribution of the combination of criteria or definitions used by the authors. This refers to 23 articles that used a definition derived by the authors.

Criteria were grouped as “Clinical”, “Micro” (Microbiology), and “Imaging” for better visualization. The Sankey diagram (Fig. 1) illustrates diagnostic category distribution, showing that most studies relied on clinical criteria alone, followed by clinical combined with microbiological or imaging data. Fewer cases used imaging alone or all three categories, highlighting a predominant reliance on clinical evaluation in PSI diagnosis.

## 4 Discussion

Our study found that published studies used variable definitions of PSI, with the most common definitions based primarily on clinical signs and symptoms. Moreover, we showed that most previous research relied on predefined criteria for PSI, such as those established by the CDC and ACS NSQIP. While this study focuses on PSIs as a general category, we acknowledge that these infections can involve various spinal compartments, each with unique clinical characteristics and implications. Most of the included studies applied the CDC surveillance definition, classifying infections as either deep or organ/space. However, the specific anatomical sites were often not differentiated, and a broad definition of infection was commonly used.

The NHSN criteria represent one of the most recent definitions for PSI, incorporating clinical features, microbiological culture results, and imaging findings. However, similarly to earlier definitions, the NHSN criteria are primarily designed for surveillance purposes and may have limited applicability in guiding clinical decision-making (Centers for Disease Control and Prevention, 2024). Relying on this definition in clinical practice may hinder timely diagnosis and limit the identification of atypical presentations. The detailed characteristics of all criteria are summarized in Table S2. While the definitions provided by CDC, NHSN, and ACS NSQIP are largely consistent, NHSN incorporates non-culture-based diagnostic methods in the classification of superficial, deep, and organ/space SSIs. Additionally, NHSN extends the postoperative surveillance period for deep and organ/space SSIs up to 90 d, offering a more comprehensive assessment of infection risk following surgery.

The proposed methodology, designed for application across various medical fields, was tested in the transition from native vertebral osteomyelitis (NVO) (Petri et al., 2024b, a) to PSI, revealing challenges. While both conditions share similarities, PSI presents unique diagnostic complexities due to its iatrogenic etiology, variable timing from the index procedure (e.g., 6 weeks vs. 90 d), and unique clinical presentation. Diagnosis of PSI is also challenging due to the frequent presence of hardware that impacts imaging performance, the differential of wound drainage, and pseudoarthrosis or hardware failure (Tai et al., 2024). The categorization of PSI definitions must balance comprehensive yet parsimonious diagnostic criteria to avoid overfitting or underfitting. Using fewer criteria may improve clarity but risk oversimplification, while too many can create an unmanageable number of combinations. Also, the different CDC definitions for SSI updated over time are not specifically tailored for spine surgery, and the distinction between superficial, deep, and organ/space infections is not clearly categorized in the studies we reviewed. Moreover, we showed that no study specifically used sonication, molecular diagnostics, or novel biomarkers to diagnose PSI.

Similarly to what has already been done for SSI (Christensen et al., 2021; Ju et al., 2015), we highlight that, in the realm of spine surgery, NHSN and NSQIP systems often yield discordant results due to differences in case identification and definitions. Previous studies have consistently shown higher SSI rates when using ACS NSQIP, likely because the NHSN does not account for outpatient SSIs (Ju et al., 2015). Also, infection rates vary depending on the diagnostic definition applied (Nota et al., 2015). These systems rely on subjective clinical decisions, which can impact consistency in PSI rates, and we also advocate for a unified definition system.

The articles included in this study covered a broad spectrum of surgical procedures, including decompression, fusion, and discectomy, performed with or without instrumentation. Only one study specifically addressed microsurgical techniques. Efforts to standardize the definition of PSI by combining these diverse surgical approaches may affect the accuracy of infection diagnosis. Challenges to implementing a unified definition – such as variability in available resources, diagnostic limitations (e.g., imaging artifacts caused by hardware), and clinician resistance to adopting new criteria – underscore the need for future prospective studies and consensus panels. These initiatives are essential to refine the definition and classification of PSI, improve diagnostic precision, and clarify the implications for both clinical practice and infection surveillance.

## 5 Conclusions

This study highlights the critical need for a clear and standardized definition of postoperative spinal infection (PSI) that integrates microbiological, radiological, and clinical factors. Current surveillance systems often fail to capture the complexity of clinical presentations, leading to diagnostic uncertainty. However, the feasibility and utility of establishing a single standardized definition for all PSIs may be questionable, given the clinical heterogeneity across surgical contexts, such as the presence of hardware, procedure type, and timing of infection. While a unified framework could risk remaining a primarily academic exercise, we propose a flexible, tiered approach that accommodates these differences and supports both clinical decision-making and research. A collaborative effort is needed to develop consensus definitions that balance scientific rigor with real-world applicability.

## Supplement

10.5194/jbji-10-451-2025-supplement The supplement related to this article is available online at https://doi.org/10.5194/jbji-10-451-2025-supplement.

## Data Availability

No data sets were used in this article.
