# Supplementary material for: Defining postoperative spinal infections: navigating the inconsistencies in diagnostic definitions
Source: J Bone Jt Infect. 2025 Nov 12;10(6):451–7. doi: 10.5194/jbji-10-451-2025 (PMC12628270; doi:10.5194/jbji-10-451-2025)
Supplement: The supplement related to this article is available online at https://doi.org/10.5194/jbji-10-451-2025-supplement. [file jbji-10-451-2025-supplement.pdf]

Supplement of J. Bone Joint Infect., 10, 451–457, 2025
https://doi.org/10.5194/jbji-10-451-2025-supplement
© Author(s) 2025. CC BY 4.0 License.

Supplement of

Defining postoperative spinal infections: navigating the inconsistencies in diagnostic definitions

Seyed Mohammad Amin Alavi et al.

Correspondence to: Elie F. Berbari (berbari.elie@mayo.edu)

The copyright of individual parts of the supplement might differ from the article licence.

Fig. S1: PRISMA flowchart

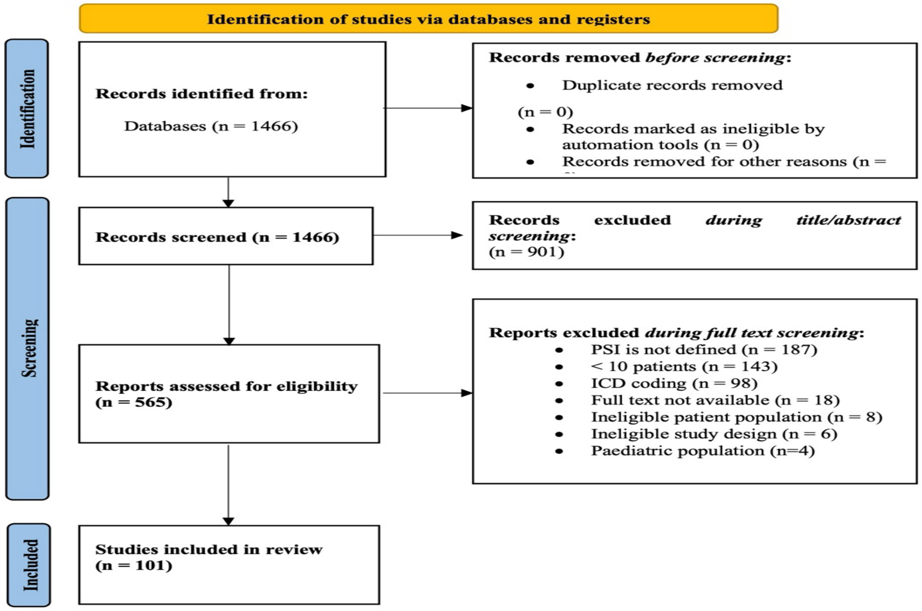

Table S1. Search strategy (Ovid MEDLINE(R) 1946 to Present and Epub Ahead of Print, In-Process & Other Non-Indexed Citations and Ovid MEDLINE(R) Daily)

#	Searches
1	exp Spine/su
2	exp Spinal Fusion/
3	exp Lumbar Vertebrae/su
4	exp Cervical Vertebrae/su
5	Spinal Stenosis/su
6	Laminectomy/
7	Intervertebral Disc Displacement/su or exp intervertebral disk hernia/su
8	exp Diskectomy/
9	(discectom* or diskectom* or microdiscectom*).ti.
10	((spine or spinal or lumbar or lumbo* or vertebr* or sacral or sacrum or trans-sacr* or sacro* or disc or discs or disk* or intradiscal or intradiskal or intervertebral*) adj3 (surg* or operation* or reoperat* or re-operat* or fuse or fused or fusion or fusing)).ti.
11	("interbody fusion*" or "inter-body fusion*").ti,ab.
12	or/1-11
13	*wound infection/
14	postoperative complications/
15	surgical wound infection/

16	(seroma* or "serous discharge" or dehiscence or infection or infected or infect or SSI or osteomyelitis).ti,ab.
17	13 or 15 or 16
18	12 and 14 and 17
19	(exp animals/ or exp nonhuman/) not exp humans/
20	((alpaca or alpacas or algae* or amphibian or amphibians or animal or animals or antelope or armadillo or armadillos or avian or baboon or baboons or bats or beagle or beagles or bee or bees or bird or birds or bison or bovine or buffalo or buffaloes or buffalos or "c elegans" or "Caenorhabditis elegans" or camel or camels or canine or canines or canis or carp or cats or catfish or cattle or chamaeleo* or chameleon* or chick or chicken or chickens or chicks or chimp or chimpanze or chimpanzees or chimps or cow or cows or "D melanogaster" or "dairy calf" or "dairy calves" or deer or dog or dogs or donkey or donkeys or drosophila or "Drosophila melanogaster" or duck or duckling or ducklings or ducks or equid or equids or equine or equines or feline or felines or ferret or ferrets or finch or finches or fish or flatworm or flatworms or fox or foxes or frog or frogs or "fruit flies" or "fruit fly" or "G mellonella" or "Galleria mellonella" or geese or gerbil or gerbils or goat or goats or goose or gorilla or gorillas or groundhog or groundhogs or hamster or hamsters or hare or hares or heifer or heifers or horse or horses or iguana or iguanas or insect or insects or jellyfish or kangaroo or kangaroos or kitten or kittens or "laboratory animal*" or lagomorph or lagomorphs or lamb or lambs or lemur or lemurs or lemuridae or llama or llamas or macaque or macaques or macaw or macaws or marmoset or marmosets or mice or minipig or minipigs or mink or minks or monkey or monkeys or mouse or mule or mules or muskrat

	or muskrats or nematode or nematodes or newt or newts or octopus or octopuses or orangutan or "orang-utan" or orangutans or "orang-utans" or oxen or parrot or parrots or pig or pigeon or pigeons or piglet or piglets or pigs or porcine or primate or primates or poultry or quail or rabbit or rabbits or rat or rats or reptile or reptiles or rodent or rodents or ruminant or ruminants or salmon or sheep or shrimp or slug or slugs or swine or tamarin or tamarins or tilapia or tilapias or toad or toads or trout or urchin or urchins or vole or voles or waxworm or waxworms or weasel or weasels or wolf or wolves or worm or worms or wrass* or xenopus or "zebra fish" or zebrafish) not (human or humans or patient or patients)).ti,ab,hw,kw.
21	(rat or rats or mice or mouse or murine or pig or pigs or porcine or swine or dog or dogs).ti.
22	or/19-21
23	18 not 22
24	limit 23 to english language

Table S2. Comparison of Definitions for Superficial Incisional, Deep Incisional, and Organ/Space Surgical Site Infections (SSI) According to Various Surveillance Guidelines.

Definition	Superficial SSI	Deep SSI	Organ/Space SSI
CDC 1992 (Horan et al., 1992)	• ≤30 days postop<br>• Involves skin/subcutaneous tissue only<br>• Requires ≥1:<br>1. Purulent drainage<br>2. Positive culture from incision<br>3. Signs of infection (pain, swelling, redness, heat) and incision opened by surgeon (unless culture negative)<br>Diagnosis by surgeon/physician	• ≤30 days postop (if no implant) OR ≤1 year (if implant in place and infection related to procedure)<br>• Involves deep soft tissues (fascial and muscle layers)<br>• Requires ≥1:<br>1. Purulent drainage from deep incision (not organ/space)<br>2. Deep incision spontaneously dehisces or deliberately opened by surgeon with fever (>38 °C), pain, or tenderness (unless culture negative)<br>3. Abscess or evidence of infection found on exam, reoperation, histopathology, or imaging<br>4. Diagnosis by surgeon/physician	• ≤30 days postop (if no implant) OR ≤1 year (if implant in place and infection related to procedure)<br>• Involves any part of the anatomy (organs or spaces) other than the incision that was opened or manipulated during surgery<br>• Requires ≥1:<br>1. Purulent drainage from a drain placed into the organ/space<br>2. Positive culture from fluid or tissue in the organ/space<br>3. Abscess or other evidence of infection found on exam, reoperation, histopathology, or imaging<br>4. Diagnosis by surgeon/physician
CDC 1999 (Mangram et al., 1999)	• ≤30 days post-op<br>• Skin or subcutaneous tissue only<br>• Requires ≥1:<br>1. Purulent drainage	• ≤30 days postop (if no implant) or ≤1 year (if implant in place and infection related to surgery)<br>• Deep soft tissues (fascial and muscle layers)	• ≤30 days postop (if no implant) or ≤1 year (if implant in place and infection related to surgery)

	2. Positive culture<br>3. Clinical signs (pain, redness, swelling, heat) + incision opened by surgeon<br>4. Diagnosis by surgeon/physician	• Requires ≥1:<br>1. Purulent drainage from deep incision (not organ/space)<br>2. Spontaneous or surgeon-opened deep incision with fever, pain, or tenderness (unless culture negative)<br>3. Abscess or evidence of infection on exam, reoperation, histopathology, or imaging<br>4. Diagnosis by surgeon/physician	• Any organ or anatomical space manipulated during the procedure, excluding the incision<br>• Requires ≥1:<br>1. Purulent drainage from a drain placed into the organ/space<br>2. Positive culture from fluid or tissue in the organ/space<br>3. Abscess or evidence of infection on exam, reoperation, histopathology, or imaging<br>4. Diagnosis by surgeon/physician
CDC 2017	Same as NHSN		
NHSN (Centers for Disease Control and Prevention, 2025)	• ≤30 days post-op<br>• Superficial incision or subcutaneous tissue only<br>• Requires ≥1:<br>1. Purulent drainage from the superficial incision<br>2. Organisms identified from aseptically obtained specimen (culture or non-culture microbiologic test)<br>3. Superficial incision deliberately opened/re-accessed by surgeon/physician designee without	• ≤30 or 90 days post-op<br>• Deep soft tissues (fascia, muscle layers) of the incision<br>• Requires ≥1:<br>1. Purulent drainage from the deep incision<br>2. Deep incision opened, re-accessed, aspirated, or spontaneously dehisced by surgeon/physician/designee, AND Organism(s) identified from deep soft tissues (culture or non-culture microbiologic test) AND patient has ≥1 symptom:	• ≤30 or 90 days post-op<br>• Involves the organ/space tissues (deeper than the fascia/muscle)<br>• Requires ≥1:<br>1. Purulent drainage from a drain placed into the organ/space<br>2. Organism(s) identified from fluid or tissue in the organ/space by a culture or non-culture based microbiologic testing method<br>3. Abscess or evidence of infection in deep incision

	testing, plus at least one sign of infection (pain, swelling, erythema, heat)<br>4. Diagnosis by physician or designee	fever >38 °C, localized pain, or tenderness<br>3. Abscess or evidence of infection in deep incision seen on exam, histopathology, or imaging	seen on exam, histopathology, or imaging
ACS-NSQIP (American College of Surgeons, 2025)	• ≤30 days post-op<br>• Only skin or subcutaneous tissue<br>• Requires ≥1:<br>1. Purulent drainage (lab confirmation optional)<br>2. Organisms from aseptically obtained culture<br>3. Clinical signs or symptoms (pain, tenderness, swelling, redness, heat) AND incision opened by surgeon (unless culture negative)<br>4. Diagnosis by surgeon/physician	• ≤30 days postop (if no implant) or ≤1 year (if implant in place and infection related to surgery)<br>• Involves deep soft tissues (fascial and muscle layers)<br>• Requires ≥1:<br>1. Purulent drainage from the deep incision (not from organ/space component)<br>2. Deep incision spontaneously dehisces or is deliberately opened by surgeon, with ≥1 of: fever (>38 °C), localized pain, or tenderness (unless culture is negative)<br>3. Abscess or evidence of infection in the deep incision found on exam, reoperation, histopathology, or imaging<br>4. Diagnosis of a deep incisional SSI by surgeon or attending physician	• ≤30 days postop (if no implant) or ≤1 year (if implant in place and infection related to surgery)<br>• Involves any part of the anatomy (organs or spaces) other than the incision that was opened or manipulated during surgery<br>• Requires ≥1:<br>1. Purulent drainage from a drain placed through a stab wound into the organ/space (excludes drains placed during the operation with continuous drainage since surgery)<br>2. Organisms isolated from an aseptically obtained culture of fluid or tissue in the organ/space<br>3. Abscess or other evidence of infection in the organ/space found on exam, reoperation, histopathology, or imaging

			4. Diagnosis of an organ/space SSI by a surgeon or attending physician

References:

American College of Surgeons. (2025). *ACS NSQIP® User Guide for the Participant Use Data File*. https://www.facs.org/quality-programs/acs-nsqip

Centers for Disease Control and Prevention. (2025). *Surgical Site Infection (SSI) Event*. https://www.cdc.gov/nhsn

Horan, T. C., Gaynes, R. P., Martone, W. J., Jarvis, W. R., & Grace Emori, T. (1992). CDC definitions of nosocomial surgical site infections, 1992: A modification of CDC definitions of surgical wound infections. *American Journal of Infection Control*, *20*(5), 271–274. https://doi.org/https://doi.org/10.1016/S0196-6553(05)80201-9

Mangram, A. J., Horan, T. C., Pearson, M. L., Silver, L. C., & Jarvis, W. R. (1999). Guideline for Prevention of Surgical Site Infection, 1999. Centers for Disease Control and Prevention (CDC) Hospital Infection Control Practices Advisory Committee. *American Journal of Infection Control*, *27*(2), 97–132; quiz 133–134; discussion 96.

References of Figure 1:

Abdul-Jabbar, A., Takemoto, S., Weber, M. H., Hu, S. S., Mummaneni, P. V, Deviren, V., Ames, C. P., Chou, D., Weinstein, P. R., Burch, S., & Berven, S. H. (2012). Surgical Site Infection in Spinal Surgery: Description of Surgical and Patient-Based Risk Factors for Postoperative Infection Using Administrative Claims Data. *Spine*, *37*(15). https://journals.lww.com/spinejournal/fulltext/2012/07010/surgical_site_infection_in_spinal_surgery_.12.aspx

Aydinli, U., Karaeminoğullari, O., & Tişkaya, K. (1999). Postoperative deep wound infection in instrumented spinal surgery. In *Acta Orthopaedica Belgica* (Vol. 65, Issue 2, pp. 182–187).

Blomstedt, G. C. (1985). Infections in neurosurgery: A retrospective study of 1143 patients and 1517 operations. *Acta Neurochirurgica*, *78*(3), 81–90. https://doi.org/10.1007/BF01808684

Breuninger, M., Yagdiran, A., Willinger, A., Biehl, L. M., Otto-Lambertz, C., Kuhr, K., Seifert, H., Fätkenheuer, G., Lehmann, C., Sobottke, R., Siewe, J., & Jung, N. (2020). Vertebral Osteomyelitis After Spine Surgery: A Disease With Distinct Characteristics. *Spine*, *45*(20). https://journals.lww.com/spinejournal/fulltext/2020/10150/vertebral_osteomyelitis_after_spine_surgery__a.11.aspx

Buser, Z., Chang, K.-E., Kall, R., Formanek, B., Arakelyan, A., Pak, S., Schafer, B., Liu, J. C., Wang, J. C., Hsieh, P., & Chen, T. C. (2022). Lumbar surgical drains do not increase the risk of infections in patients undergoing spine surgery. *European Spine Journal*, *31*(7), 1775–1783. https://doi.org/10.1007/s00586-022-07130-0

Chang, C.-W., Tsai, T.-T., Niu, C.-C., Fu, T.-S., Lai, P.-L., Chen, L.-H., & Chen, W.-J. (2019). Transforaminal Interbody Debridement and Fusion to Manage Postdiscectomy Discitis in Lumbar Spine. *World Neurosurgery*, *121*, e755–e760. https://doi.org/https://doi.org/10.1016/j.wneu.2018.09.211

Fernandez, M. C., Gottlieb, M., & Menitove, J. E. (1992). Blood transfusion and postoperative infection in orthopedic patients. *Transfusion*, *32*(4), 318–322. https://doi.org/https://doi.org/10.1046/j.1537-2995.1992.32492263444.x

Glassman, S. D., Dimar, J. R., Puno, R. M., & Johnson, J. R. (1996). Salvage of instrumental lumbar fusions complicated by surgical wound infection. *Spine*, *21*(18), 2163–2169. https://doi.org/10.1097/00007632-199609150-00021

Goel, V., Kaizer, A., Patwardhan, A. M., Ibrahim, M., DeSimone, D. C., Sivanesan, E., & Shankar, H. (2022). Postoperative Oral Antibiotic Use and Infection-Related Complications After Spinal Cord Stimulator Surgery. *Neuromodulation*, *25*(5), 738–744. https://doi.org/10.1016/j.neurom.2021.10.012

Khalooeifard, R., Oraee-Yazdani, S., & Vahdat Shariatpanahi, Z. (2022). Obesity and posterior spine fusion surgery: A prospective observational study. *International Journal of Orthopaedic and Trauma Nursing*, *45*, 100920.

https://doi.org/https://doi.org/10.1016/j.ijotn.2021.100920

Kobayashi, K., Imagama, S., Ito, Z., Ando, K., Yagi, H., Hida, T., Ito, K., Ishikawa, Y., Tsushima, M., & Ishiguro, N. (2017). Is a Drain Tip Culture Required After Spinal Surgery? *Clinical Spine Surgery*, *30*(8). https://journals.lww.com/jspinaldisorders/fulltext/2017/10000/is_a_drain_tip_culture_required_after_spinal.4.aspx

Korge, A., Fischer, R., Kluger, P., & Puhl, W. (1994). The importance of sonography in the diagnosis of septic complications following spinal surgery. *European Spine Journal*, *3*(6), 303–307. https://doi.org/10.1007/BF02200141

Kudo, Y., Okano, I., Toyone, T., Matsuoka, A., Maruyama, H., Yamamura, R., Ishikawa, K., Hayakawa, C., Tani, S., Sekimizu, M., Hoshino, Y., Ozawa, T., Shirahata, T., Fujita, M., Oshita, Y., Emori, H., Omata, H., & Inagaki, K. (2020). Lateral lumbar interbody fusion in revision surgery for restenosis after posterior decompression. *Neurosurgical Focus FOC*, *49*(3), E11. https://doi.org/https://doi.org/10.3171/2020.6.FOCUS20361

Kutlay, M., Colak, A., Simsek, H., Yildiz, S., Topuz, K., Kaya, S., Cetinkal, A., & Demircan, M. (2008). Antibiotic and hyperbaric oxygen therapy in the management of post-operative discitis. *Undersea & Hyperbaric Medicine : Journal of the Undersea and Hyperbaric Medical Society, Inc*, *35*(6), 427–440.

Li, Y.-D., Chi, J.-E., Chiu, P.-Y., Kao, F.-C., Lai, P.-L., & Tsai, T.-T. (2021). The comparison between anterior and posterior approaches for removal of infected lumbar interbody cages and a proposal regarding the use of endoscope-assisted technique. *Journal of Orthopaedic Surgery and Research*, *16*(1), 386. https://doi.org/10.1186/s13018-021-02535-x

McCormack, R. A., Hunter, T., Ramos, N., Michels, R., Hutzler, L., & Bosco, J. A. (2012). An Analysis of Causes of Readmission After Spine Surgery. *Spine*, *37*(14). https://journals.lww.com/spinejournal/fulltext/2012/06150/an_analysis_of_causes_of_readmission_after_spine.12.aspx

Molina, E., Zhao, D., Dowlati, E., Carroll, A. H., Mueller, K. B., Sandhu, F. A., & Voyadzis, J.-M. (2021). Minimally invasive posterior lumbar surgery in the morbidly obese, obese and non-obese populations: A single institution retrospective review. *Clinical Neurology and Neurosurgery*, *207*, 106746. https://doi.org/https://doi.org/10.1016/j.clineuro.2021.106746

Park, M.-S., Moon, S.-H., Kim, H.-S., Hahn, S.-B., Park, H.-W., Park, S.-Y., & Lee, H.-M. (2006). A Comparison of Autologous and Homologous Transfusions in Spinal Fusion. *Yonsei Med J*, *47*(6), 840–846. https://doi.org/10.3349/ymj.2006.47.6.840

Pull ter Gunne, A. F., & Cohen, D. B. (2009). Incidence, prevalence, and analysis of risk factors for surgical site infection following adult spinal surgery. *Spine*, *34*(13), 1422–1428. https://doi.org/10.1097/BRS.0b013e3181a03013

Shifflett, G. D., Bjerke-Kroll, B. T., Nwachukwu, B. U., Kueper, J., Burket, J., Sama, A. A., Girardi, F. P., Cammisa, F. P., & Hughes, A. P. (2016). Microbiologic profile of infections in presumed aseptic revision spine surgery. *European Spine Journal*, *25*(12),

3902–3907. https://doi.org/10.1007/s00586-016-4539-8

Spatenkova, V., Bradac, O., Jindrisek, Z., Hradil, J., Fackova, D., & Halacova, M. (2021). Risk factors associated with surgical site infections after thoracic or lumbar surgery: a 6-year single centre prospective cohort study. *Journal of Orthopaedic Surgery and Research*, *16*(1), 265. https://doi.org/10.1186/s13018-021-02418-1

Tavares-Júnior, M. C. M., Cabrera, G. E. D., Teixeira, W. G. J., Narazaki, D. K., Ghilardi, C. S., Marcon, R. M., Cristante, A. F., & Barros-Filho, T. E. P. de. (2021). Risk Factors Associated with Postoperative Infection in Cancer Patients Undergoing Spine Surgery. *Clinics*, *76*, e2741. https://doi.org/https://doi.org/10.6061/clinics/2021/e2741

Tronnier, V., Schneider, R., Kunz, U., Albert, F., & Oldenkott, P. (1992). Postoperative spondylodiscitis: Results of a prospective study about the aetiology of spondylodiscitis after operation for lumbar disc herniation. *Acta Neurochirurgica*, *117*(3), 149–152. https://doi.org/10.1007/BF01400612
